# Acculturation stress and allostatic load among Mexican immigrant women *

**DOI:** 10.1590/1518-8345.2578.3135

**Published:** 2019-04-29

**Authors:** Karen Therese D’Alonzo, Frances Munet-Vilaro, Dennis P. Carmody, Peter J. Guarnaccia, Anne Marie Linn, Lisa Garsman

**Affiliations:** 1 Rutgers, The State University of New Jersey , School of Nursing , Newark , NJ , EUA .; 2 California State University-Monterey Bay , Department of Nursing , Seaside , CA , EUA .; 3 Rutgers, The State University of New Jersey , Department of Human Ecology , New Brunswick , NJ , EUA .; 4 St. Peter’s University , School of Nursing , Jersey City , NJ , EUA .

**Keywords:** Allostasis, Acculturation, Obesity, Immigrants, Metabolic Syndrome, Case-Control Study, Allostasis, Aculturação, Obesidade, Imigrantes, Síndrome Metabólica, Estudo Caso-Controle, Allostasis, Aculturación, Obesidad, Inmigrantes, Síndrome Metabólico, Estudio Caso-Control

## Abstract

**Objectives:**

this case-control study compared levels of stress and allostatic load (AL) among Mexican women in the US ( *n* =19) and Mexico ( *n* = 40).

**Method:**

measures of stress included the Perceived Stress Scale (PSS) and the Hispanic Women’s Social Stressor Scale (HWSSS). A composite measure of 8 indicators of AL (systolic and diastolic blood pressure, body mass index (BMI), waist-to-hip ratio, total cholesterol, glycated hemoglobin (hemoglobin A1C), triglycerides and C-reactive protein) was calculated.

**Results:**

there were no significant group differences in AL between Mexican and Mexican immigrant women ( *t* = 1.55, *p* = .126). A principal component factor analysis was conducted on the 8 AL indicators; a 2-factor solution explained 57% of the variance. Group differences in the two AL factors were analyzed using MANOVA. BMI and waist-to-hip ratios were lower, but blood pressure and triglycerides were higher in the US group and were mediated by time in the US. Greater acculturation stress was significantly related to increased waist-to-hip ratio ( *r* = .57, *p* = .02).

**Final remarks:**

findings suggest some measures of AL increased with time in the US, and acculturation stress may be a significant factor.

## Abstract (pt)

**Objetivos:**

este estudo de caso-controle comparou os níveis de estresse e carga alostática (CA) entre mulheres mexicanas nos EUA ( *n* = 19) e no México ( *n* = 40).

**Método:**

medidas de estresse incluíram a Escala de Estresse Percebido (EEP) e a Escala de Estresse Social das Mulheres Hispânicas (EESMH). Uma medida composta por 8 indicadores de CA (pressão arterial sistólica e diastólica, índice de massa corporal (IMC), relação cintura-quadril, colesterol total, hemoglobina glicada (hemoglobina A1C), triglicerídeos e proteína C-reativa) foi calculada.

**Resultados:**

não houve diferenças significativas entre os grupos na CA entre mulheres mexicanas imigrantes e não imigrantes ( *t* = 1,55, *p* = 0,126). Uma análise fatorial de componentes principais foi realizada nos 8 indicadores de CA; uma solução de 2 fatores explicou 57% da variância. As diferenças entre grupo nos dois fatores CA foram analisadas usando MANOVA. O IMC e a relação cintura-quadril foram menores, mas a pressão arterial e os triglicerídeos foram maiores no grupo dos EUA e foram mediados pelo tempo nos EUA. O maior estresse de aculturação foi significativamente relacionado ao aumento da relação cintura-quadril ( *r* = 0,57, *p* = 0,02).

**Considerações finais:**

os resultados sugerem que algumas medidas de CA aumentam com o tempo nos EUA e o estresse de aculturação pode ser um fator significativo.

## Abstract (es)

**Objetivos:**

este estudio de casos y controles ha comparado los niveles de estrés y carga alostática (CA) en mujeres mexicanas en los EE.UU. ( *n* = 19) y México ( *n* = 40).

**Método:**

las medidas de estrés incluyeron la Escala de Estrés Percibido (EEP) y la Escala de Estrés Social de las Mujeres Hispanas (HWSSS, por sus siglas en inglés). Se calculó una medida compuesta de 8 indicadores de CA (presión arterial sistólica, presión arterial diastólica, índice de masa corporal (IMC), relación cintura/cadera, colesterol total, hemoglobina glicosilada (HbA1c), triglicéridos, y proteína C reactiva).

**Resultados:**

no hubo diferencias significativas de CA entre los grupos de mujeres mexicanas e inmigrantes mexicanas ( *t* = 1,55, *p* = 0,126). Se realizó un análisis factorial de componente principal de los 8 indicadores de CA; una solución de 2 factores explicó el 57% de la varianza. Las diferencias de grupo en los dos factores de CA se analizaron utilizando MANOVA. El IMC y la relación cintura/cadera disminuyeron, pero la presión arterial y los triglicéridos aumentaron en el grupo de los EE.UU. y estuvieron influenciados por el tiempo de residencia en los EE.UU. Un mayor estrés aculturativo se relacionó significativamente con el aumento de la relación cintura/cadera (r = 0,57, p = 0,02).

**Comentarios finales:**

los hallazgos sugieren que algunas medidas de CA aumentan con el tiempo de residencia en los EE.UU. y el estrés aculturativo puede ser un factor importante.

## Introduction

The US and Mexico are neighbors sharing the longest and most active migration corridor in the world. More than 11.7 million Mexican immigrants currently reside in the United States ^(^ ^1^ ^)^ comprising the largest immigrant group in the country. Although migration from Mexico has slowed greatly in the past few years ^(^ ^1^ ^)^ , Mexico still has a greater percentage of its citizens living abroad (mostly in the US) than any other country in the world ^(^ ^2^ ^)^ . Moreover, more than one-third of these *mexicanos en el extranjero* (Mexicans living abroad) have resided in the US for 15 years or more, many with precarious legal status ^(^ ^3^ ^)^ .

Consistent with the Hispanic Paradox ^(^ ^4^ ^-^ ^5^ ^)^ data, the risk for cardiovascular disease among immigrant Latinos is relatively low at the time of arrival but increases greatly with length of residence in the US ^(^ ^6^ ^)^ . Mexican-American women in particular have one of the world’s highest rates (44%) of metabolic syndrome (MS), a disorder characterized by central (abdominal) obesity, insulin resistance/hyperinsulinemia, hypertension, and dyslipidemia ^(^ ^7^ ^)^ , all of which increase the risk for atherosclerotic disease.

Using a framework of allostatic load (AL) ^(^ ^8^ ^)^ , one factor that may contribute to MS among Mexican immigrant women is perceived stress ^(^ ^9^ ^)^ . Acculturation-related factors such as family separation, cultural conflicts, low socioeconomic status, language barriers, racism and discrimination and low perceived control over employment may contribute to chronic stress and may predispose Mexican immigrant women to the development of MS. Recently, the threat of deportation is a major stressor for many families where one or more members are among *los sin papeles* (the undocumented). To date, there is little known regarding patterns of AL accumulation and the impact of chronic stress on AL among Mexican immigrant women ^(^ ^10^ ^)^ .

In its simplest form, acculturation can be conceptualized as a normative process that occurs when a person from one culture is exposed to another culture ^(^ ^11^ ^)^ . In contrast, acculturation stress is defined as a negative reaction to intercultural contact or the cultural adaptation process. Mexican immigrants to the US may experience acculturation stress when seeking housing, work, or education or because of racial/ethnic discrimination and loss of social support ^(^ ^12^ ^)^ . These conflicts are frequently encountered by new immigrants; however, if migration does not result in a substantially higher quality of life and financial security, acculturation stress may become a chronic state ^(^ ^13^ ^)^ . This is particularly true among the undocumented. Acculturation stress has long been associated with poor mental health in a number of studies among Mexican immigrants ^(^ ^14^ ^-^ ^15^ ^)^ . Consistent with the model of (AL), recent studies suggest that cumulative exposures to high levels of chronic psychological stressors may lead to a variety of physiologic conditions as well.

**Figure 1:**
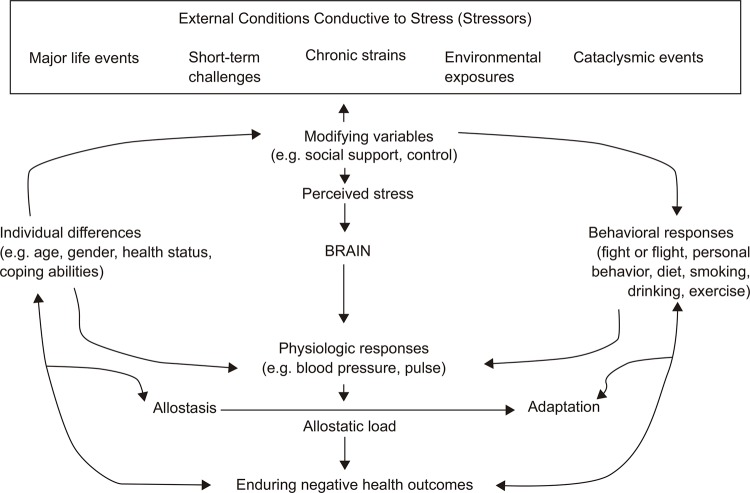
The stress response and development of allostatic load

Source: Adapted from McEwen (1998) and Israel and Schuman (1990). Adapted with permission from Massachusetts Medical Society, © 1998 and from Jossey-Bass, respectively.

The biobehavioral process of AL ( Figure 1 ) provides a compelling framework to explain the link between cumulative exposure to chronic psychological and physiological stressors and the prevalence of chronic illnesses among minority groups. Allostasis is the bodily mechanism by which humans and other organisms cope with short-term physiological and psychological stress. Similar to acculturation, it is a normative process. However, the allostatic process may become ineffective if the stress itself persists over an extended period of time, the body does not recognize the stressor as having been resolved, or the body’s mechanisms for shutting off the stressor are not functioning ^(^ ^16^ ^)^ . AL is the collective term used to refer to damage incurred by the body as it adapts to such psychosocial or physical stressors. It has been posited that AL may negatively affect the body through a variety of biochemical mechanisms ^(^ ^8^ ^)^ . Constant exposure to frequent stress may lead to unexplained surges in blood pressure, the overproduction of stress-related hormones resulting in gradual degradation of the immune system, and “overloading” of the body’s compensatory mechanisms. Over time, these physiological responses to chronic stress can manifest through myocardial infarction, stroke, hypertension, type 2 diabetes mellitus, and certain types of cancers.

AL is generally measured through a composite index of signs and symptoms of cumulative strain on various organs and tissues, with a concentration on the cardiovascular system ^(^ ^17^ ^)^ . Systolic and diastolic blood pressures, total cholesterol, triglycerides, fasting glucose, glycated hemoglobin (hemoglobin A1C), body mass index (BMI), waist circumference and waist-to-hip ratio serve are examples of some of the biomarkers used to assess AL. Individuals with a high AL are particularly prone to develop truncal weight gain, insulin insensitivity and other characteristics of MS. Although the terms AL and MS are sometimes used interchangeably, the data suggest that they are distinct concepts ^(^ ^18^ ^)^ ; MS can best be understood as one manifestation of AL.

There is a limited body of knowledge regarding AL among Mexicans and Mexican immigrants. In an early study of AL among Mexicans, Mexican immigrants had lower AL scores upon arrival than US-born Mexican Americans, non-Hispanic whites, and non-Hispanic blacks, and this advantage lessened with duration of residence in the US ^(^ ^6^ ^)^ . The authors controlled for a number of demographic, socioeconomic, and health input covariates but did not measure perceived stress directly. Similarly, in a study of adults in Texas, foreign-born Mexicans had lower AL scores than US-born Mexican Americans, and acculturation measures did not account for the difference ^(^ ^19^ ^)^ . Chronic work-related, financial, and caregiving stressors were associated with elevated ALs in a separate study, but immigrant women were not part of the sample ^(^ ^9^ ^)^ . To date, few studies have focused on the impact of perceived stress associated with acculturation as a factor in AL among Mexican immigrant women.

This study compared Mexican women from the indigenous state of Oaxaca who immigrated to the US with a matched group of women born and residing in Oaxaca using measures of perceived stress and a range of biomarkers of AL, including body mass index (BMI), waist-to-hip ratio, systolic and diastolic blood pressure, and finger stick collections of dried blood spots (DBSs) for C-reactive protein, HgbA1C, total cholesterol and triglycerides. Four hypotheses were proposed: 1) Levels of AL among immigrant Mexican women in the US will be higher than women in Mexico; 2) Length of time living in the US will predict levels of AL; 3) Perceived stress scores will be greater among women living in the US; and 4) Acculturation stress, but not perceived stress, will predict levels of AL among women in the US.

## Methods

A matched case-control design was used in this pilot study. Approval for the study was granted by the Institutional Review Board of Rutgers University, Rutgers Biomedical and Health Sciences (Pro 20140000012) and the Vice-Rector of the Universidad de la Sierra Sur campus of the State University of Oaxaca in Miahuatlán, Mexico.

Data collection took place in two locations: 1) an urban community of approximately 56,000 residents in central New Jersey, where 40-50% of the full-time residents of the community are immigrant families from southern Mexican states, particularly Oaxaca; and 2) a small indigenous community of approximately 14,000 residents in the western Sierra Sur region of Oaxaca, which is the home to a large percentage of these immigrants. Participants were recruited in both the US and Mexico using purposive sampling methods. In the US, *promotoras* /trained community health workers first sought out women who immigrated from the specific community in Oaxaca through personal contacts and announcements placed in church bulletins. Later, in Mexico, a matched sample of women was recruited through the local Health Center/Centro de Salud by a local female physician who was well regarded by the women in the community. Participants were matched by age. Many of the women from the Oaxaca (control) sample were considered blood relatives (e.g., first and second cousins) of the women in the US (case) sample; the use of relatives as controls helped to assure the groups had significant overlap and helped to limit confounding by genetic factors thought to be related to MS and elevated AL ^(^ ^20^ ^)^ . The 1:2 sampling ratio was chosen based on published recommendations for case-control studies ^(^ ^21^ ^)^ . Likewise, it has been noted that when the number of cases in a study is small, the ratio of controls to cases can be raised to improve the ability to find significant differences ^(^ ^22^ ^)^ . The sample size of 59 women is consistent with the number of subjects required for pilot studies using a comparative design to calculate effect sizes to estimate power and sample size needed for a larger study ^(^ ^23^ ^)^ . Sample sizes of 15-25 per group were recommended for small to medium standardized effect sizes ^(^ ^24^ ^)^ .

Subjects were all premenopausal adult women born in the state of Oaxaca, between 18-45 years of age, nonpregnant, and able to understand Spanish and/or English. Menopausal and postmenopausal women were excluded because of the greater prevalence of MS in this population.

Participants were enrolled using verbal consent since the lead author’s previous experience working in the immigrant Mexican community ^(^ ^25^ ^)^ suggested that many indigenous Mexican women are comfortable speaking in Spanish but much less comfortable reading and writing in Spanish. Participants provided demographic information regarding age, number of years of education, marital status, residence of spouse (US or Mexico), number of pregnancies and living children, place of birth and when applicable, the number of years living in the US. Spanish language versions of the questionnaires were used for both groups; bilingual research assistants (Mexico) and *promotoras* (US) assisted participants by reading items to them when necessary. All participants completed a 14-item Spanish language version of the Perceived Stress Scale (PSS) ^(^ ^26^ ^)^ . This version ^(^ ^27^ ^)^ measured the degree to which social situations were appraised as stressful. It is widely used and has utility in predicting biomarkers of stress. Individual scores on the PSS can range from 0 to 56 with higher scores indicating higher perceived stress. Prior to use, the literacy level of the PSS and the Hispanic Women’s Social Stressor Scale (HWSSS) ^(^ ^28^ ^)^ were assessed using the Fernandez-Huerta method ^(^ ^29^ ^)^ , and both were found to be at a 7 ^th^ grade level. The PSS has been tested among adults in Mexico with a reported internal consistency among women of α = .78 ^(^ ^30^ ^)^ . The HWSSS, a 41-item scale used to assess social stressors experienced by Mexican and Mexican-American women, was chosen for this study from among other scales as a proxy measure of acculturation stress. This decision was made in consultation with a group of community *promotoras* ; the majority of acculturation stress scales reviewed focused on the experiences of new immigrants (particularly English language proficiency issues). In contrast, the HWSSS appeared to more adequately address the stressors of immigrant women who had been living in the US for a decade or more. The 41 items are based, in part, on items derived from the Immigrant version of the Hispanic Stress Inventory (HSI), a well-established tool to measure acculturation stress ^(^ ^31^ ^)^ . Individual scores on the HWSSS can range from 0 to 123, with higher scores indicating higher perceived stress. In preliminary testing, the authors identified six subscales of the HWSSS (Immigration, Socioeconomic, Racism, Familial, Parental and Unemployment); Cronbach’s alpha coefficients ranged from .73-.94 ^(^ ^28^ ^)^ .

The aforementioned measures of AL were assessed. DBSs were selected because they are a minimally invasive method of incorporating biomarkers into population-based health research in a binational study. SurgiLance Pink SLN 300, 2.88 mm 21-gauge high-flow lancets were used to obtain the finger-stick specimens. After discarding the first drop of blood, a drop was placed in each of the five circles on a Whatman 903™ Protein Saver Card. In keeping with the protocol established by the Biomolecular Laboratory at the University of Rochester, School of Nursing, the samples were given adequate time to dry and then stored in low gas-permeability plastic bags with a desiccant packet added to reduce humidity. When the specimens were sufficiently dry, the blood spots were a dark brownish color and no bright red areas were visible ^(^ ^32^ ^)^ . The packets were then mailed to the laboratory from collection sites in both the US and Mexico. Elution and analyses of the DBS eluates were carried out in the laboratory using a standardized process ^(^ ^33^ ^)^ . Participants in the US were compensated with a $10 gift card to a popular “big-box” store. Following consultation with local leaders, participants in Mexico were given a bag of groceries containing rice, beans and tuna fish.

Following the methods of Seeman et al. ^(^ ^34^ ^)^ , a measure of allostatic load (AL) was designed to summarize levels of physiological activity across a range of regulatory systems, including systolic and diastolic blood pressure, BMI and waist-to-hip ratio, total cholesterol, triglycerides and HgbA1c. For each of the 8 indicators of AL, participants were classified into quartiles based on the distribution of scores of the entire cohort. AL was the sum of the number of indicators where the participant was in the highest risk quartile. In this scoring system, AL scores could range from 0-8. Table 1 shows the cutoff points used for each AL component. The absence of multivariate outliers was examined by calculating Mahalanobis distances for each participant. Outlier participants were identified by comparing the calculated value to the critical chi square value at *p* = .001 with 4 degrees of freedom. One participant had a Mahalanobis distance that exceeded the criterion and was excluded from the MANOVA analyses. Levene’s test was used to test for homogeneity of variance.

**Table 1:** Cutoff points for allostatic load (AL) indicators used for serum biomarker samples collected in the cities of New Brunswick, NJ, USA and Santa Maria Zacatapec, Oaxaca, Mexico, 2015

Biological parameters	Highest risk quartile
Systolic blood pressure (mmHg)	≥120
Diastolic blood pressure (mmHg)	≥ 80
Body Mass Index (kg/m ^2^ )	≥ 33.30
Waist-to-hip ratio	≥ .93
Total cholesterol (mg/dL)	≥271.45
Hemoglobin A1C(%)	≥5.88
Triglycerides (mg/dL)	≥161.51
C-reactive protein (mg/L)	≥3.85

While the original AL model was based on a single factor ^(^ ^8^ ^)^ , more recent research has suggested that a two-factor model might explain more of the variance in AL ^(^ ^35^ ^)^ . More recently, it has been suggested that model invariance across subpopulations does not preclude the possibility that the measurement of AL may differ importantly by the studied sample ^(^ ^35^ ^)^ . Therefore, the one-factor and two-factor models need to be examined in this study to determine which model is a better fit to the data in the sample.

Descriptive statistics were computed for all variables. Next, the eight components of AL were examined by factor analysis to determine whether a single-factor or a two-factor model was a better fit to the data.

For hypothesis 1, the AL scores for the two groups (US and Mexico) were compared by an independent t–test. Multivariate analysis of variance (MANOVA) was used to identify the specific indicators that led to group differences in AL and to build successive models to identify covariates that explained the differences. Wilk’s lambda was used to assess whether the MANOVAs were significant. Hypothesis 2 was tested using Pearson’s r to identify the specific AL indicators that were associated with length of time living in the US. Mediational analysis was then conducted with the indicator regressed on years in the US and with PSS scores as a mediator ^(^ ^36^ ^)^ . For hypothesis 3, independent t-tests were used to test for group differences (US and Mexico) in perceived stress (PSS). For hypothesis 4, Pearson’s r was used to examine the bivariate correlations between the PSS and AL and the HWSSS and AL. Analyses were conducted using SPSS Version 23, and the significance threshold for all analyses was set at *p* =.05.

## Results

A total of 19 immigrant women were enrolled in the study in the US. These cases were then matched with 40 women of the same age and indigenous background (Tacuate) who resided in the home community in Mexico. The ages of women in both countries were similar (US: M =36.46, SD = 8.28; Mexico: M = 32.65, SD = 6.58). Ninety percent of the women in the US were married, as were 75% of the women in the Mexico sample. Women living in the US had a mean 3.05 children (SD = 1.08), while Mexican women had a mean 2.57 children (SD = 1.70). Forty-two percent of women in the US finished the equivalent of elementary school in Mexico, while another 42% listed middle school as their highest level of education. While a similar percentage of women in Mexico attended only elementary school (40%), another 27.5% went on to graduate from high school, and 17.5% attended or graduated from college. As a community that experiences high levels of emigration, 87.5% of the women in Oaxaca reported having friends or relatives who had immigrated to the US, and 17% reported that their spouse was currently living in the US. All of the women in the US lived with their spouses. The mean time in the US was 16.37 years (range 5-22 years, SD = 4.92). Scores on both the PSS and the HWSSS were normally distributed (Shapiro-Wilk values for PSS = .99 and for the HWSSS = .94; *p* values were *p* = .88 and *p* = .28, respectively). Cronbach’s alpha for the PSS was .71 and .95 for the HWSSS. The means and standard deviations of the study variables (n = 59) are presented in Table 2.

**Table 2:** Means (M), standard deviations (SD) of study variables of the sample of women (n = 59) in the cities of New Brunswick, NJ, USA, and Santa Maria Zacatapec, Oaxaca, Mexico, 2015

Variable	Country	M*	SD ^†^
Total AL ^‡^ score	Mexico US ^§^	1.95 2.58	1.90 1.35
BMI ^||^ (kg/m ^2^ )	Mexico US ^§^	31.28 27.65	5.46 3.15
SBP ^¶^ (mm Hg)	Mexico US ^§^	101.79 124.11	13.55 19.98
DBP ** (mm Hg)	Mexico US ^§^	71.54 81.68	10.39 12.67
Waist-to-hip ratio	Mexico US ^§^	.94 .82	.15 .03
TC ^††^	Mexico US ^§^	213.36 233.36	57.69 57.99
Hemoglobin A1c (%)	Mexico US ^§^	5.26 5.36	.92 .70
TG ^§§^ (mg/dl)	Mexico US ^§^	122.21 163.54	46.72 78.78
CRP ^||||^ (mg/L)	Mexico US ^§^	3.15 2.52	3.21 2.12
PSS ^¶¶^	Mexico US ^§^	33.27 30.89	5.79 4.88
HWSSS***	Mexico US ^§^	N/A 52.00	N/A 17.50

* M – Mean; †SD: Standard deviation; ‡AL: Allostatic load; §US-United States; ||BMI - Body mass index; ¶SBP - Systolic blood pressure; ** DBP - Diastolic blood pressure; ††TC - Total cholesterol; §§TG – Triglycerides; ||||CRP - C-reactive protein; ¶¶PSS - Perceived Stress Scale; ***HWSSS - Hispanic Women’s Social Stressor Scale

The eight indicators of AL were examined by two principal component factor analysis with Promax rotation and Kaiser normalization. In the first analysis, the solution was set to one factor. The single factor explained 35% of the variance. A second analysis was conducted by setting the solution to two factors. The two factors explained 57% of the variance. The first component (cardiovascular) explained 35% of the variance and consisted of systolic and diastolic blood pressure, total cholesterol and triglycerides. The second component (metabolic) explained an additional 22% and consisted of BMI, waist-to-hip ratio, hemoglobin A1C and C-reactive protein.

*Group Differences in AL* (Hypothesis 1). The AL scores for the two groups (US and Mexico) were first compared by an independent t-test. The AL score for the US group ( *M* = 2.58, *SD* = 1.35) was not significantly different than the AL score of the Mexican group ( *M* = 1.95, *SD* = 1.50), *t* (57) = 1.55, p = .13. However, the US women had lower BMI and waist-to-hip ratios but significantly higher systolic and diastolic blood pressure and triglycerides (TG) than the women in Mexico. The result demonstrates that the combined AL score did not differ between groups. Separate MANOVAs were then conducted for each factor (cardiovascular and metabolic). The first MANOVA examining the cardiovascular indicators of AL found a statistically significant difference between the groups, *F* (4, 51) = 7.719, *p* < .001; Wilk’s Λ = 0.623, partial η ^2^ = .377. Levene’s test was not significant, indicating homogeneity of variance. As shown in Table 3, the women in the US had significantly higher levels of DBP, SBP and TG than women living in Oaxaca.

**Table 3:** Group differences in cardiovascular indicators of AL (N = 59) of the sample of women in the cities of New Brunswick, NJ, USA, and Santa Maria Zacatapec, Oaxaca, Mexico, 2015

Indicator	F value (1, 56 df*)	p value ^†^	η ^2^	US ^‡^ (M ^§^ , SE ^||^ )	MEX ^¶^ (M ^§^ , SE ^||^ )
DBP**	10.46	.002	.162	81.68, 2.60	71.35, 1.86
SBP ^††^	25.63	.001	.322	124.10, 3.65	101.35, 2.62
TC ^‡‡^	1.55	.218	.028	233.48, 13.18	213.27, 9.44
TG ^§§^	6.04	.017	.10	163.54, 13.63	122.35, 9.77

*df - degrees of freedom; † *p* value - significance test for group differences; ‡US - United States; §M – Mean; ║SE - Standard error of the estimate; ¶MEX – Mexico; **DBP: Diastolic blood pressure; ††SBP: Systolic blood pressure; ‡‡TC - Total cholesterol; §§TG - Triglycerides

The second MANOVA found a statistically significant difference between groups in the metabolic indicators of AL, *F* (4, 51) = 11.50, *p* < .001; Wilk’s Λ = 0.526, partial η ^2^ = .474. Levene’s test was significant for the indicators of BMI and waist-to-hip ( *p* <.05) indicating a lack of homogeneity of variance. Examining the ANOVAs for each indicator revealed significant group differences for 2 of the indicators. As shown in Table 4, women in the US had lower BMI and waist-to-hip ratios than women living in Mexico. Using a nonparametric approach, both BMI, ( *U = 1.84,* z = 2.77, *p* = .006), and waist-to-hip, ( *U = 47,* z = 5.17, *p* < .001) showed significant group differences.

**Table 4:** Group differences in metabolic indicators of allostatic load (AL) (N = 59) of the sample of women in the cities of New Brunswick, NJ, USA, and Santa Maria Zacatapec, Oaxaca, MX, 2015

Indicator	F value (1, 54 df*)	p value ^†^	η ^2^	US ^‡^ (M ^§^ , SE ^||^ )	MEX ^¶^ (M ^§^ , SE ^||^ )
BMI ** (kg/m ^2^ )	6.62	.013	.109	27.69, 1.15	31.28, 0.79
CRP ^††^ (mg/L)	0.56	.458	.010	2.46, 0.71	3.10, 0.49
HemoglobinA1c(%)	0.12	.735	.002	5.37, 0.21	5.29, 0.14
Waist-to-hip ratio	47.12	<.001	.466	0.83, 0.01	0.92, 0.01

*df - degrees of freedom; † *p* value - significance test for group differences; ‡US - United States; §M – Mean; ||SE - Standard error of the estimate; ¶MEX - Mexico; **BMI - Body Mass Index; ††CRP - C-reactive protein

*Length of time in the US and AL (* Hypothesis 2 *).* The MANOVAs were repeated with the covariates of age and education levels. The same group differences were found, and the same 5 indicators were different between groups. Given the hypothesis that acculturation stress is related to AL, a covariate was added to the 2 MANOVAs. The group differences in the cardiovascular factor were no longer significant when the covariate of years in the US was added ( *F* (4, 51) = 0.74, *p* = .566; Wilk’s Λ = 0.945, partial η ^2^ = .055). Levene’s test was not significant, indicating homogeneity of variance. In addition, there were no group differences in the indicators of DBP, SBP, and TG when accounting for years of residence in the US. This finding suggests that time in the US is associated with specific indicators of AL, specifically systolic and diastolic blood pressure and triglycerides. To examine the effect of time in the US on indicators of AL, a mediational analysis was conducted with systolic blood pressure regressed on years in the US and with Perceived Stress Scale (PSS) scores as a mediator ^(36)^ . The overall model was significant, *F* (2, 54) = 10.27, *p* < .001, *R* ^2^ = .32, Time in the US had a significant direct effect on SBP (β *=1.33, t* = 4.51, *p <* .001 *, 95% CI,* 0.73, 1.92 *)* with no indirect effect through PSS (β *=-.005, t* = -.008, *p =* .99). Similar patterns were found for both diastolic blood pressure and total triglycerides. The conclusion is that time in the US is directly and proportionately associated with increased SBP, DBP and TG and PSS scores do not mediate the associations.

*Group differences in perceived stress scores (Hypotheses 3).* The perceived stress scores for the two groups (US, Mexico) were compared by an independent t-test. Perceived stress scores were higher among women in Mexico ( *M* = 33.27, *SD* = 5.79) than the US ( *M* = 30.89, *SD* =4.88), but not significantly so ( *t* (57) =-1.52, *p* =.13). This finding suggests that there were no differences in perceived stress between Mexican and Mexican immigrant women in the study.

*Relationships among perceived stress, acculturative stress and AL (Hypothesis 4).* In the US group, acculturation stress scores (HWSSS) were not correlated with total AL scores but were significantly correlated with the AL indicator waist-to-hip ratio ( *r* = .57, *p* = .02). In the US group, PSS was not significantly correlated with total AL scores or any of the 8 indicators of AL. Among women in the US, total scores of the PSS were not significantly correlated with total scores of the HWSSS ( *r* = .21, *p* = .52), supporting the assertion that the instruments addressed two distinct phenomena, perceived stress (PSS) and acculturative stress (HWSSS). The small sample size of women in the US ( *n* = 19) precluded the use of regression analyses. Nonetheless, these findings suggest that there are unique acculturation-related stressors that may play a significant role in promoting truncal weight gain among this group of Mexican immigrant women. Specific items on the HWSSS that caused considerable stress for the US women included living with relatives; being ignored or getting poor service at stores or offices because they were Hispanic; not having enough money to pay for necessities such as food for their families or shoes for their children; feeling lonely and isolated and missing the help and support of their family in Mexico.

## Discussion

Our findings support previous studies ^(^ ^6^ ^,^ ^18^ ^)^ , which linked AL among Mexican immigrants with increased time spent in the US. This relationship was particularly true in this study with regard to systolic and diastolic blood pressure and triglycerides. Given that the participants in the study were young to middle-aged women, the prevalence of hypertension in the US group was somewhat unexpected, although the results of other studies indicate that hypertension is likely underdiagnosed and untreated among immigrant Latinos ^(^ ^37^ ^)^ .

Despite the higher scores for blood pressure and triglycerides, we did not find a significant difference in AL between the two groups of women. This was likely due to the unexpectedly higher BMI and waist-to-hip ratios among women in the Mexican group. Although acculturation stress has traditionally been thought to contribute to weight gain among immigrant women ^(^ ^38^ ^-^ ^40^ ^)^ , an additional factor that may parallel this process among Mexican women is the nutrition transition. The nutrition transition is defined as a broad shift in dietary habits and physical activity that coincides with economic, demographic, and epidemiological changes ^(^ ^38^ ^)^ . For the past twenty years, Mexico has been in Stage 4 of the nutrition transition, which is a phase characterized by weight gain and an increase in nutrition-related noncommunicable diseases. In contrast, the US is shifting into Stage 5, where the focus is on weight loss/weight management and behavioral changes. As a result, it is quite possible that Mexican immigrants who arrive in the US in the near future may be heavier than their counterparts in the US, a finding that challenges the so-called Hispanic Paradox. Further analyses of dietary intake among both groups of women may help to confirm these findings.

Although the number of years spent in the US was associated with increases in selected indicators of AL, the length of time more closely approximates acculturation, not acculturation stress. Empirical evidence suggests that it is not the acculturation process itself but the stress of adapting to life in a new country that has the greatest impact on the physical and emotional health of Latino immigrants. Perceived stress scores were slightly higher among women living in Mexico than in the US. This finding is not totally unexpected; the very fact that such a high percentage of Oaxacans immigrate to the US suggests that life is financially and emotionally difficult for those who stay behind ^(^ ^39^ ^-^ ^40^ ^)^ . However, the presence of a significant association between a specific source of stress, namely, acculturation, and waist-to-hip ratio, provides preliminary support for the AL model among Mexican immigrant women.

This binational pilot study took a novel approach to assess the effect of acculturation stress on AL among Mexican women from the same community living in the US and Mexico. In contrast to previous studies that simply linked acculturation to AL using a proxy measure of the number of years living in the US, our study used two distinct measures to quantify the relationship between stress and AL. In contrast to previous studies, our US sample contained a significant percentage of women who had lived as a *mexicana en el extranjero* for more than a decade, a situation that is becoming more common. Long-term immigrants suffer from a unique set of challenges that have not been well addressed in the literature ^(^ ^41^ ^)^ . The two groups of women were well matched regarding age and indigenous background. This study sets the stage for a larger investigation into the effects of acculturation stress on AL in Mexican immigrant women, which could potentially inform a multipronged intervention to lower AL in this group.

There are a number of limitations to the present study. The use of purposive sampling limits the generalizability of the findings. A significant number of the women in the US sample did not work outside the home and had not experienced instances of work-place discrimination; many lived in close proximity to each other, which may have offered some protection for them against acculturation stress, or the so-called “barrio advantage” ^(^ ^42^ ^)^ . The unique reciprocal relationship between the two binational communities also posed some interesting challenges, including the issue of return migrants to Mexico. Following the recession in the late 2000s, a significant percentage of Mexican immigrants from the US returned to Mexico, convinced they could make a better living in their homeland. The Mexican community we visited was no exception. When immigrants return to rural Mexico, they often create a demand for lifestyles they adopted in the US ^(^ ^43^ ^)^ . These preferences include building fast food restaurants and a greater reliance on automobiles as a means of transportation. These changes affect the community at large and may add to the obesogenic environment that characterizes Stage 4 of the nutrition transition in Mexico. On a related note, we did not directly measure physical activity and nutritional intake in this study; these two variables would add greatly to our understanding of the role that stress plays in AL and should be included in future studies. In this study, we limited our choice of biomarkers to measures that could easily be collected in a community setting. This decision precluded us from assessing neuroendocrine markers such as 24-hour urinary norepinephrine or epinephrine. In addition, the laboratory we used was not able to accommodate DBS analysis for inflammatory biomarkers such as interleukin6 (IL-6) and tumor necrosis factor alpha (TNF-α). Focus groups may be needed to explore the potential for collecting additional biomarkers. Finally, it can be argued that the sample size (N = 59) was too small to conduct the two principal component factor analyses. There is no consensus in the factor analysis literature concerning a minimum sample size. While older references suggest there should be at least 10 cases per variable, with a total of at least 100 cases ^(^ ^44^ ^)^ , other more recent sources recommend limiting the number of variables and factors to assure moderate to high levels of communality ^(^ ^45^ ^)^ . Following the more recent guidelines, we restricted the number of variables to 8 and the number of factors in our final model to 2. In our study, communalities ranged from .43 to .75, placing it just below the .6 to .8 average recommended as a high range ^(^ ^45^ ^)^ . It has been suggested that small sample sizes may not be a problem when the data are highly reliable and communality levels are high ^(^ ^46^ ^)^ . Accordingly, we had highly reliable measures of biomarkers (systolic BP, HgbA1C, total cholesterol, triglycerides, and waist-to-hip ratio), and our communality levels were moderate to high. Lastly, we achieved convergence using Promax rotation as a solution. Often, samples that are too small will fail to converge ^(^ ^47^ ^)^ . For these reasons, we argue that principal component factor analysis was appropriate in our study.

## Conclusion

In summary, this study supports evidence of a link between length of residence in the US and acculturation stress in some indicators of AL among Mexican immigrant women. To attenuate the declines in health status among Mexican immigrants postulated by the Hispanic Paradox, further research will be needed to clarify the roles played by acculturation and acculturation stress in the genesis of allostatic load.
